# CMRI-detected brain injuries and clinical key risk factors associated with adverse neurodevelopmental outcomes in very preterm infants

**DOI:** 10.1038/s41598-025-02539-1

**Published:** 2025-05-25

**Authors:** Karla Drommelschmidt, Thomas Mayrhofer, Hanna Müller, Borek Foldyna, Janika Raudzus, Sophia L. Göricke, Bernd Schweiger, Selma Sirin

**Affiliations:** 1 Department of Pediatrics I, Neonatology, Pediatric Intensive Care, and Pediatric Neurology, University Hospital Essen, University of Duisburg-Essen, Hufelandstraße 55, 45147 Essen, Germany; 2 Center for Translational Neuro- and Behavioral Sciences (cTNBS), University Hospital Essen, University of Duisburg-Essen, Essen, Germany; 3 School of Business Studies, Stralsund University of Applied Sciences, Zur Schwedenschanze 15, 18435 Stralsund, Germany; 4 Department of Radiology Cardiovascular Imaging Research Center, Massachusetts General Hospital, Harvard Medical School, 55 Fruit Street, Boston, MA 02114 USA; 5 Division of Neonatology, Department of Pediatrics, University Hospital of Tuebingen, Calwerstraße 7, 72076 Tuebingen, Germany; 6 Department of Diagnostic and Interventional Radiology and Neuroradiology, University Hospital Essen, University of Duisburg-Essen, Hufelandstraße 55, 45147 Essen, Germany; 7 Department of Diagnostic Imaging, University Children’s Hospital Zurich University of Zurich, Steinwiesstraße 75, Zurich, 8032 Switzerland

**Keywords:** Pediatric research, Brain injuries, Magnetic resonance imaging, Neurodevelopmental outcomes, Preterm infants, Diseases, Health care, Medical research, Neurology, Risk factors

## Abstract

Neurological impairment is high after preterm birth. This study evaluates the impact and interplay of cMRI-detected brain injuries (BI) and clinical risk factors on neurodevelopmental outcomes and extracts the most important key factors. A retrospective analysis was conducted on risk factors (perinatal/neonatal, cMRI-detected BI) for adverse motor (MO) and cognitive (CO) outcomes (Bayley Scales of Infant Development, 24 months corrected age) in a tertiary center cohort (2009–2018) of very preterm infants (< 32 weeks of gestation) using uni-/multivariable regression models. We included 342 infants (mean gestational age:28.0 ± 2.3 weeks; male:49%). Significant clinical predictors for MO/CO included GA, birthweight, APGAR score, catecholamine treatment, ventilation, retinopathy of prematurity, transfusion of red blood cells (RBCs), bronchopulmonary dysplasia, surgery, and patent ductus arteriosus interventions (all *p* < 0.01/*p* < 0.01), surfactant (MO: *p* = 0.037), and sepsis (*p* < 0.001/*p* = 0.016). (Severe) cMRI-detected BIs (> 1, all *p* < 0.05) and not only severe intraventricular hemorrhage (IVH) III°/III°+PVHI and ventricular dilatation (VD) (all *p* < 0.05), but also mild/moderate injuries like IVH II° (*p* < 0.001/*p* < 0.024), cerebellar hemorrhage (CO: *p* = 0.028), and moderate VD (MO: *p* = 0.005) significantly impacted outcomes. Independent key factors were > 1 severe cMRI-detected BI (MO/CO:-11.27/-10.3 score points (sp), *p* = 0.021/0.043), APGAR score (10 min, MO/CO:+5.3/+4.45 sp/point, *p* < 0.001/*p* < 0.001), surfactant administration (MO:+4.88 sp, *p* = 0.031), and transfusion of RBCs (MO/CO:-1.69/-1.96 sp/transfusion, *p* = 0.006/*p* < 0.001). In conclusion, combining imaging and clinical (key) risk factors is important for risk stratification of preterm infants. Even mild BI, like IVH II°, significantly contributes to adverse outcomes, underlining the importance of cMRI.

**Supplementary Information:**

The online version contains supplementary material available at 10.1038/s41598-025-02539-1.

## Introduction

The worldwide rate of preterm births is rising and has reached 10.6%^1^, with European rates ranging from 4.2–8.3%^2^. As the survival rates of very preterm infants (born below 32 weeks of gestation) and particularly extremely preterm infants have improved^3^, there has been a notable increase in infants facing adverse neurodevelopmental outcomes^4–6^. In Germany, the preterm birth rate has been reported to be 8.0%, while the rate for very preterm infants is 1.43% (2017–2020^7^). Approximately 50% of the very preterm infants and, particularly extremely preterm infants (< 28 weeks of gestation), experience long-term neurodevelopmental impairment^4,8^, characterized by motor and cognitive impairments with an inverse correlation between severity of impairment and gestational age^8,9^. This represents a substantial burden on individuals and society, persisting frequently throughout childhood, adolescence, and into adulthood^10^.

Several established risk factors contribute to adverse neurodevelopmental outcomes in preterm infants, encompassing perinatal and neonatal clinical parameters as well as brain injuries (BI) identified by neuroimaging modalities. For many years, cMRI has been used as a valuable neuroimaging tool to predict adverse outcomes in preterm infants^11,12^, as recently highlighted by Inder et al.^13^. While severe BIs (sBI) such as high-grade intraventricular hemorrhage (IVH) are known to have an impact on outcomes, the impact of moderate or low-grade BIs such as IVH II° or ventricular dilatation remains subject to debate in the literature^14,15^. Moreover, the importance of detected BIs to the entire field of clinical perinatal and neonatal risk factors for adverse neurodevelopmental outcomes such as gestational age (GA), Apgar score, bronchopulmonary dysplasia (BPD), surfactant treatment, invasive and non-invasive ventilation, patent ductus arteriosus (PDA), and inflammatory conditions (e.g., sepsis, necrotizing enterocolitis (NEC)) requires further clarification^16–22^.

Understanding the impact, the respective roles, and the interplay of risk factors in the field of perinatal medicine, spanning initial treatment in the delivery room and Neonatal Intensive Care Unit (NICU) hospitalization, as well as performing cMRI at TEA to detect BIs, is crucial for identifying infants at risk. Moreover, gaining insight into key factors for motor and cognitive impairment holds significance for clinical decision-making, parental counseling, and aiding clinicians in employing future neuroprotective strategies to enhance outcomes in preterm infants. This study aims to evaluate the impact of risk factors and extract the most influential key factors (perinatal, neonatal risk factors, and cMRI-detected BI) on neurodevelopmental outcomes in a well-defined large cohort of very preterm infants over 10 years. This knowledge enables the early initiation of neurorehabilitative support and facilitates structured post-care planning.

## Methods

### Study design

We conducted a retrospective observational cohort study assessing cMRI and clinical data among all infants born below 32 + 0 weeks of gestation, treated in the Department of Neonatology of the University Hospital Essen in Germany (level III NICU) between 01.01.2009 and 31.12.2018. The present study was approved by the Institutional Review Board and Ethics Committee of the Medical Faculty of Duisburg-Essen in accordance with the Declaration of Helsinki (ID: 12-4981-BO), and the need to obtain informed consent was waived due to the retrospective character by the Institutional Review Board and Ethics Committee of the University Duisburg-Essen.

Inclusion criteria for this study were: (1) in-house birth and treatment at the University Hospital Essen until discharge (and readmission at TEA) or TEA, (2) survival until TEA, and (3) available neurodevelopmental outcomes at a corrected age of 24 months in our outpatient clinic. Exclusion criteria were: (1) known or suspected genetic disorders or congenital infections due to an overlap in neurological symptoms and (2) cMRI at TEA is unavailable (lack of parental consent).

### Perinatal and neonatal factors

Clinical factors were collected from the medical records: GA, birthweight, birth mode (vaginal delivery, Cesarean section (primary/secondary/emergency)), multiples, sex, percentile (birthweight), small for gestational age (SGA), catecholamine treatment, Apgar score 1/5/10 minutes (point score/increase/decrease of point scores), surfactant treatment (local protocol for surfactant administration: For preterm infants < 28 + 0 weeks of gestation, administration within the first hour of life is recommended during initial care, for preterm infants ≥ 28 + 0 weeks, administration within the first 2 h of life and fractional concentration of oxygen in inspired air (FiO₂) of 30%), sepsis, NEC (Bell stage ≥ 2 ^23^/surgery), BPD^18^ (infants born < 32 weeks, who require supplemental oxygen for at least 28 days and at 36 weeks postmenstrual age), retinopathy of prematurity (ROP^24^, PDA (presence/intervention/pharmacological treatment), surgery, transfusion of red blood cells (RBCs, requirement/quantity), preterm premature rupture of membranes (rupture of gestational membranes < 37 weeks, PPROM^25^, invasive/CPAP (continuous positive airway pressure) ventilation (days).

### Magnetic resonance imaging protocol and analysis

**Fig. 1:**
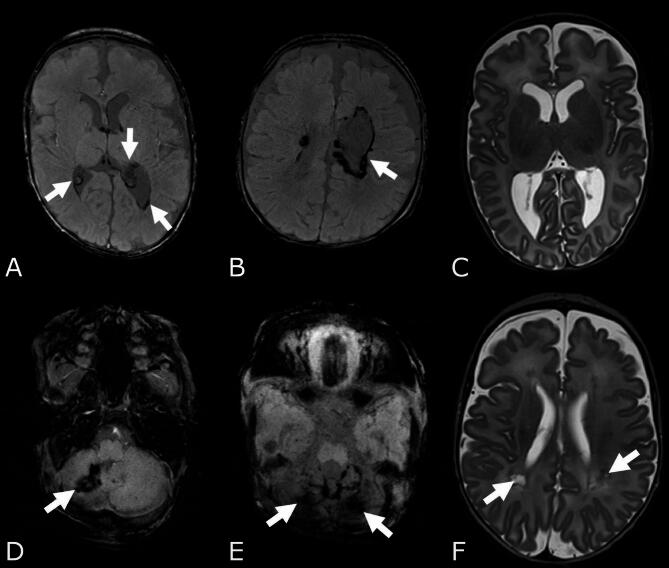
Overview: severe brain injuries. (A) IVH III° (SWI-sequence, white arrows), (B) PVHI (SWI-sequence), (C) Ventricular dilatation (T2-sequence), (D) CBH III° (SWI-sequence) (E) CBH IV° (SWI-sequence, F) bilateral cPVL (T2-sequence). *CBH*: cerebellar hemorrhage, *cPVL*: cystic periventricular leukomalacia, *IVH*: intraventricular hemorrhage, *PVHI*: periventricular hemorrhagic infarction.

CMRI was performed on a 3 Tesla scanner (*n* = 260), if possible with an MR-compatible incubator with neonatal head coil or 1.5 Tesla scanners (*n* = 82), routinely performed without sedation using our routine imaging protocol (T2 TSE transversal, 3D T1- T1-weighted imaging (fast low-angle shot (FLASH)), sagittal, susceptibility-weighted imaging (SWI), and diffusion-weighted imaging (DW/DTI)) as previously described^26,27^. If necessary, additional sequences were added. Qualitative and quantitative MR image analyses were independently performed by two pediatric radiologists (S.S. and B.S.), blinded to clinical data, accompanied by a neonatologist (K.D.). In case of discrepancies, the diagnosis was determined by consensus. A detailed description of the incidence, definition, and classification of brain injuries, including the evaluation modality, references, and illustrative examples, can be found in the previously published paper^27^. A detailed description of the incidence, definition, and classification of brain injuries, including the evaluation modality, references, and illustrative examples, can be found in the previously published paper^27^. We evaluated the presence, frequency, and severity of IVH I°-III°^28^, periventricular hemorrhagic infarction (PVHI^29^, cerebellar hemorrhages (CBH^12^, diffuse excessive high signal intensity (DEHSI^30^, ventricular dilatation (VD^31^), punctate white matter lesions (PWML^32^, uni-/bilateral and number of lesions/side), and cystic periventricular leucomalacia (cPVL^12^, uni-/bilateral). We defined brain injury (BI) and severe brain injury (sBI) as follows: BI (IVHI°-III°, PVHI, moderate/severe VD, CBH, PWML, cPVL) and sBI (IVH III°, PVHI, CBH III°+ IV°, severe VD, cPVL, Fig. 1). We grouped IVH III° and PVHI (comprising 2 cases of IVH II° with parenchymal hemorrhage and 2 cases of IVH III° with parenchymal hemorrhage) under the category of severe hemorrhages.

### Neurodevelopmental outcomes

All included infants participated in a follow-up program as part of the clinical routine. The Bayley Scales of Infant Development (BSID II: *n* = 187, III: *n* = 155) were performed by physicians, therapists, and psychologists. We defined cognitive (CO) and motor outcomes (MO) according to Goeral et al.^33^. In cases where a patient had a valid test result for either a motor or cognitive outcome, we used the available results. Briefly, the BSID score comprises motor and cognitive milestones, with higher values indicating better outcomes. Outcomes were defined as follows: favorable outcomes: ≥85 (developmental quotient > 1 SD below the mean), < 85 (moderate), and < 70 (severe) adverse outcomes.

### Statistical analysis

Continuous data are presented as mean ± standard deviation (SD) and median and interquartile range (IQR). Comparisons of continuous variables were performed using the Wilcoxon rank-sum test comparing two groups or the Kruskal-Wallis test for more than two groups. To assess the relationship of clinical, and BI parameters to MO and CO, we used univariable and multivariable ordinary least-squares linear regression models. First, significant variables were identified in univariable analyses. These associations were further interrogated in four multivariable models: Model 1 included all perinatal variables that were statistically significant in univariable analyses; Model 2 included all significant neonatal variables; Model 3 included all significant BI variables. Finally, model 4 included all significant variables from models 1 to 3. For variables that were statistically significant but highly (auto-)correlated with each other, e.g., Apgar at 1, 5, and 10 min, we chose the variable with the highest model fit in univariable analysis based on the R^2^. Due to the exploratory character of this study, the inference was guided with a two-sided 5% false positive error rate without adjustment for multiple comparisons and clinically meaningful effect sizes. All statistical analyses were performed using Stata 16.1 (StataCorp LP, College Station, Texas).

## Results

### Baseline demographic data

**Fig. 2:**
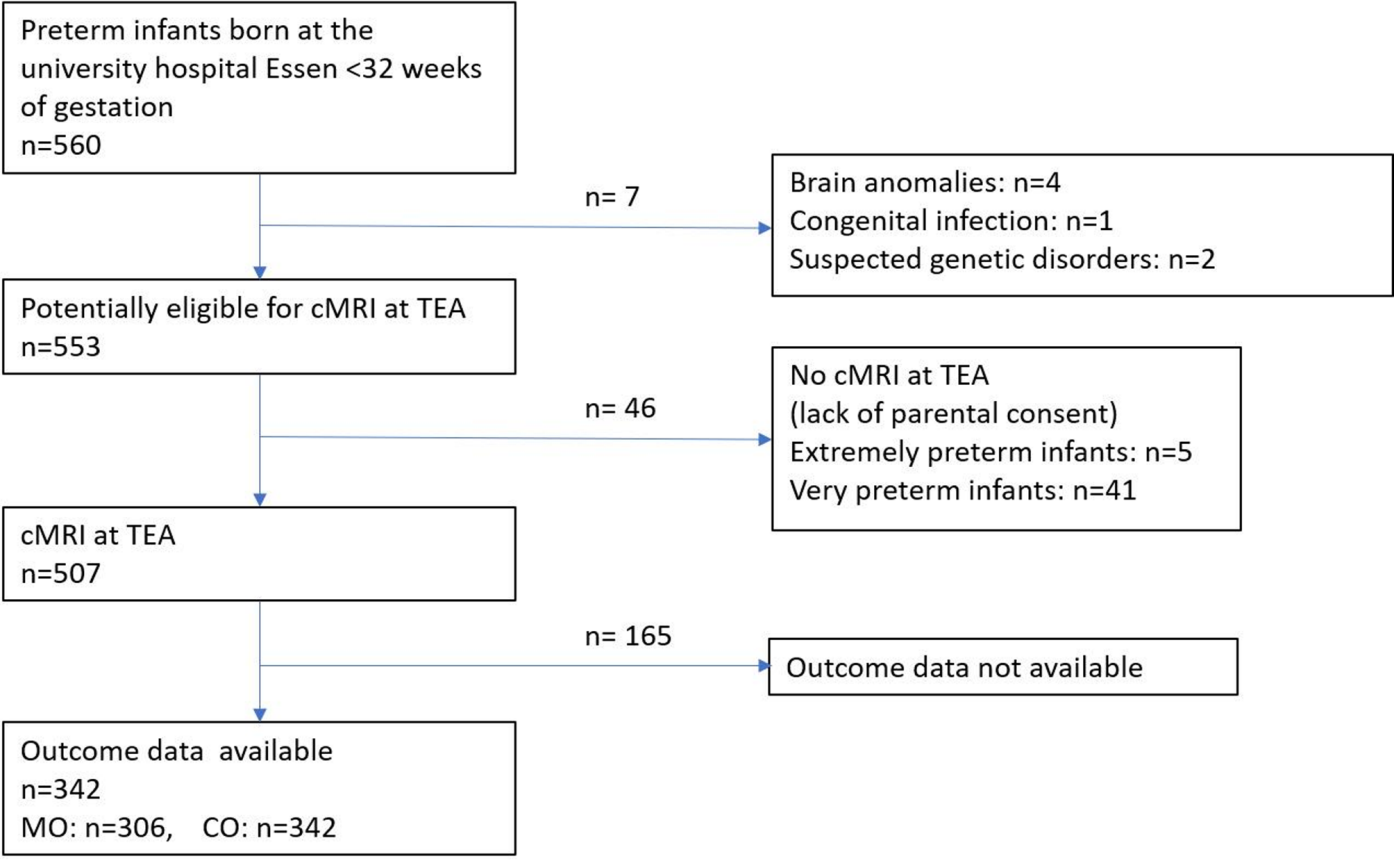
Flowchart of the study population. CO: cognitive outcome, MO: motor outcome.

The study population is presented in Fig. 2. We included 342 of 560 consecutively born infants (GA: 28.0 ± 2.3 weeks, range: 23–31 weeks, male: 168/49%), with neurodevelopmental assessment at a corrected age of 25 months (750 ± 133.7 d). CO was available for all 342 infants (93.0 ± 19.4, range: 50–152), and MO for 306 infants (88.0 ± 19.3, range: 45–137). CMRI was performed at a mean age of 40 + 1 weeks of gestation (IQR: 0–4) and was evaluable in all infants.

### Descriptive analysis to identify risk factors for adverse neurodevelopmental outcomes

#### Perinatal risk factors

A lower GA and birthweight were associated with significantly lower score points (sp) for cognitive and motor outcomes (all *p* < 0.001). SGA infants had a worse motor outcome (*p* = 0.025). Higher Apgar scores were related to better outcomes scores: infants with Apgar scores (5 min) of 0–5 scored 80.56 ± 13.49 for cognitive outcome, compared to infants with Apgar scores of 6–10 (93.32 ± 19.39, *p* = 0.026). Surfactant administration in the delivery room was associated with significantly higher motor outcome scores (MO: 90.41 ± 17.44 vs. 85.82 ± 20.71, *p* = 0.043). Catecholamine treatment was associated with significantly lower score points (MO: 70.19 ± 20.86 vs. 89.34 ± 18.57, CO: 75.96 ± 21.86 vs. 94.21 ± 18.60, both: *p* < 0.001). No significant correlation was detected for percentile (CO), sex, delivery mode, multiple births, PPROM, admission temperature, emergency cesarean section, Apgar 5 (CO)/10 minutes (Table 1).

#### Neonatal risk factors

Sepsis (MO/CO: *p* < 0.001/*p* = 0.022), ROP, and BPD (both MO/CO: *p* < 0.001/*p* < 0.001) were associated with adverse outcomes. The number of transfusions of RBCs demonstrated an inverse correlation with the neurodevelopmental outcomes (MO/CO: *p* < 0.001/*p* < 0.001). Administering 6–10 transfusions was associated with lower sp for MO (55.50 ± 10.77 vs. 93.6 ± 16.53) and CO (65.68 ± 15.10 vs. 96.84 ± 17.49). The number of invasive and CPAP ventilation days led to lower outcomes scores in infants related to ventilation duration (invasive ventilation days: MO/CO: *p* < 0.001/<0.001, CPAP days: MO/CO: *p* = 0.002/0.002). Any surgery (MO/CO: *p* < 0.001/*p* < 0.001) and PDA requiring intervention (MO: 65.67 ± 16.10; CO: 75.65 ± 19.37; PDA (pharmacological treatment: MO: 86.46 ± 19.77, CO:92.45 ± 19.49), all groups *p* < 0.001) were linked to lower outcomes scores. The presence of NEC and PDA were not associated with adverse outcomes (Table 2).

#### CMRI-detected BI

**Fig. 3:**
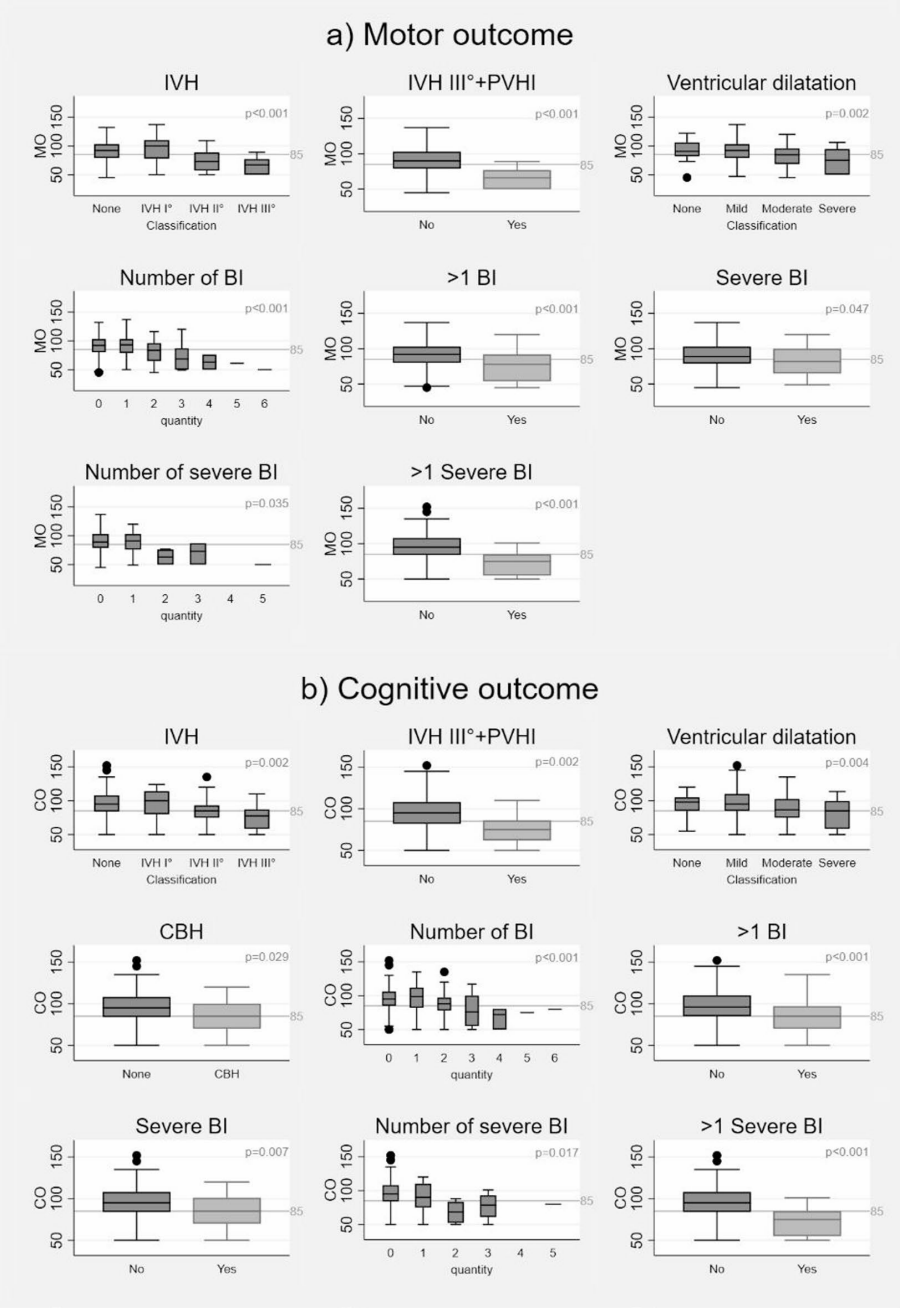
Association between cMRI-detected brain injuries and neurodevelopmental outcomes. *For motor outcome*: IVH grades, IVH III°+PVHI, ventricular dilatation, number of BI, > 1 BI, severe BI, number of severe BI, > 1 severe BI. *For cognitive outcome*: IVH grades, IVH III°+PVHI, ventricular dilatation, CBH, number of BI, <1BI, severe BI, number of severe BI, > 1 severe BI. *BI*: brain injury, *CBH*: cerebellar hemorrhage, *cPVL*: cystic periventricular leucomalacia, *IVH*: intraventricular hemorrhage, *PVHI*: periventricular hemorrhagic infarction, *PWML*: punctate white matter lesions, *sBI*: severe brain injury, *VD*: ventricular dilatation, *BI*: IVH I°; II°; III°, PVHI, moderate/severe VD, CBH I°-IV°, PWML, cPVL) and *severe BIs*: IVH III°, PVHI, CBH III°+IV°, severe VD, cPVL, significant: *p* < 0.05.

The presence of IVH was associated with adverse cognitive and motor outcomes (MO/CO: *p* < 0.001/0.002). CBH was associated with lower outcome scores for cognition (85.57 ± 21.75 vs. 93.85 ± 18.90, *p* = 0.029) but not for motor outcome (*p* = 0.093). More severe grades of ventricular dilatation were significantly associated with adverse outcomes (MO/CO: *p* = 0.002/*p* = 0.004). A higher number of BIs (MO/CO: *p* < 0.001/*p* < 0.001) and > 1 BIs (MO: 76.63 ± 21.02, CO: 83.43 ± 19.99, *p* < 0.001/*p* < 0.001) were significantly associated with adverse outcomes. The presence (MO/CO: *p* = 0.047/*p* = 0.007) and number of sBI (MO/CO: *p* = 0.035/*p* = 0.017), and the presence of > 1 sBI (MO: 63.90 ± 13.81, CO: 71.88 ± 16.95, both *p* < 0.001) were associated with adverse outcomes. No association was found for PVHI, cPVL, PWML (≥ 6/≥10), and DEHSIs (Table 3/Fig. 3).

### Multivariable models to identify risk and key factors for adverse neurodevelopmental outcomes

#### Perinatal risk factors (Model 1)

The significant parameters of the univariable analysis (GA, birthweight (< 1000 g), Apgar score, surfactant, catecholamine treatment, only MO: percentiles, and SGA, all *p* < 0.05) were analyzed by multivariable analysis. GA, SGA, Apgar score, surfactant administration, and catecholamine treatment were independent risk factors. SGA infants had significantly lower motor outcomes sp (-8.34 sp, *p* = 0.006) compared to non-SGA infants. Catecholamine treatment was also significantly associated with lower outcomes sp (MO/CO: -14.45/-15.9 sp, *p* = 0.002/*p* = 0.001). Factors linked to significantly higher scores included higher GA (MO: 2.72 sp/week of gestation, *p* = 0.001), an increase of 1 Apgar point at 10 min (MO/CO: 5.61/4.9 sp, *p* < 0.001/*p* < 0.001), and surfactant administration (MO: 5.53 sp, *p* = 0.016, Table 4, long version Supplemental Table 1).

#### Neonatal risk factors (Model 2)

Out of the significant factors of the univariable analysis (sepsis, CPAP, ROP, transfusion of RBCs, BPD, surgery, ventilation, PDA intervention, all *p* < 0.02) multivariable analysis evaluated transfusion of RBCs and ROP as risk factors. Requiring transfusions of RBCs was associated with lower motor and cognitive outcomes (MO/CO: -1.73/-1.87 sp per transfusion, *p* = 0.034/*p* = 0.012). The presence of ROP was linked to a higher risk of adverse motor outcome (-5.07 sp, *p* = 0.038, Table 4, Supplemental Table 1).

#### CMRI-detected BI as risk factors (Model 3)

Univariable analysis revealed that the following risk factors were significant: IVH II°, IVH III°, IVH III°+PVHI, PVHI (MO), VD moderate (MO), VD severe, VD moderate/severe, > 1 BI/sBI (all *p* < 0.05). The multivariable analysis detected > 1 BI and > 1 sBI as independent risk factors. The presence of > 1 BI was associated with lower motor outcome scores (MO: -8.8 sp, *p* = 0.016). The presence of > 1 sBI was associated with lower motor and cognitive outcomes scores (MO/CO: -20.19/-18.35 sp, *p* = 0.021/*p* = 0.041, Table 4, Supplemental Table 1).

#### Key factors of model 1–3 (Model 4)

**Fig. 4:**
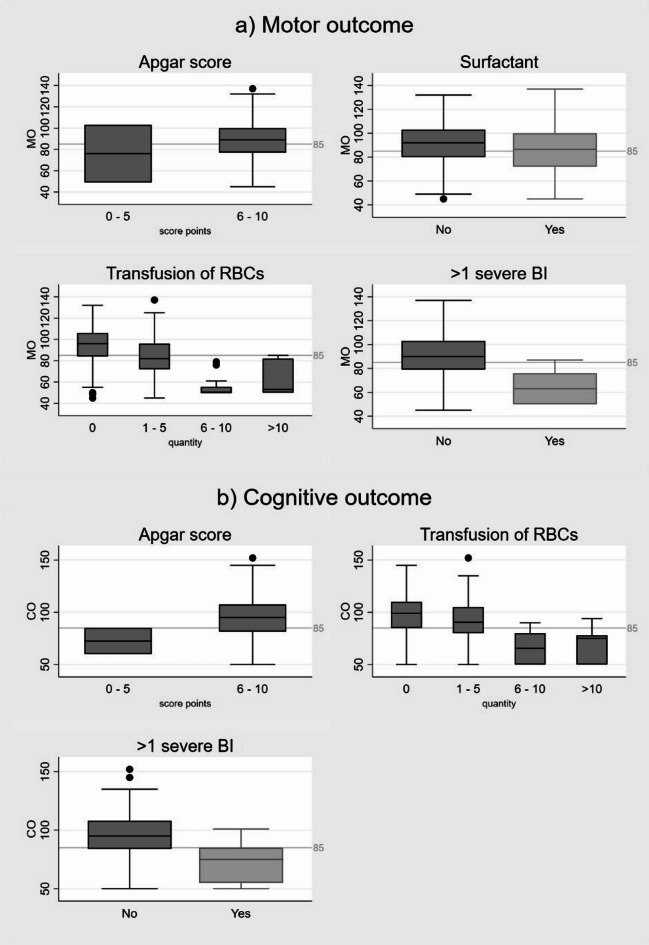
Key factors for motor and cognitive outcome of preterm infants born < 32 weeks of gestation. *For motor outcome*: (1) Apgar score at 10 min (*p* = 0.615), (2) Surfactant application in the delivery room (*p* = 0.43), (3) transfusion of RBCs (*p* < 0.001), distribution and quantity of transfusion of RBCs are shown, (4) more than one severe brain injury (*p* < 0.001). *For cognitive outcome*: (1) Apgar score at 10 min (*p* = 0.153), (2) transfusions of RBCs (*p* < 0.001), the numeric distribution and quantity of transfusion of RBCs are shown in the figure, and (3) presence of more than one severe brain injury. Descriptive values of the key factors were used for the figure. *Please note*: Surfactant application was associated with a negative coefficient in the descriptive analysis (shown in the figure) and in the multivariable regression analysis the coefficient became positive.

Model 4 included all significant variables from models 1 to 3. Key factors were the significant factors of Model 4 (Fig. 4). Higher Apgar scores at 10 min were associated with higher outcomes scores (an increase of 1 Apgar score point was linked to an increase of 5.3 sp for motor and 4.45 sp for cognitive outcomes, both *p* < 0.001). The administration of surfactant was associated with higher scores for motor outcome (4.88 sp, *p* = 0.031). Requiring a transfusion of RBCs significantly lowered outcomes scores (MO/CO: -1.69/-1.96 sp per transfusion, *p* = 0.006/*p* < 0.01). The presence of > 1 sBI lowered motor (MO/CO: -11.27/-10.3 sp, *p* = 0.021/*p* = 0.43) and cognitive outcomes (-10.3 sp, *p* = 0.043, Table 4/Fig. 4, Supplemental Table 1).

## Discussion

In this study, we identified a high number of significant risk factors that can lead to impaired cognitive and motor outcomes in a large cohort of 342 very preterm infants at a corrected age of two years, including perinatal and neonatal risk factors and cMRI-detected brain injuries. Four independent key factors for adverse neurodevelopmental outcomes were identified (the presence of more than one severe brain injury on cMRI, Apgar score, transfusion of RBCs, and surfactant application), underscoring the significance of each analyzed risk domain and its pivotal role in comprehending the impact and interplay of these factors.

Outcome prediction is crucial for identifying high-risk infants and mitigating or preventing neurological impairment^13^. Due to advancements in obstetrics and neonatology, severe neurological disabilities have declined, but infants often suffer from a wide range of mild cognitive and motor impairments that are challenging to predict^13,34^. While numerous studies have explored risk factors influencing the neurodevelopmental outcomes of preterm infants^35–37^, to our knowledge, none have integrated perinatal and neonatal risk factors with cMRI-detected brain injuries to identify key risk factors of the entire field of perinatal medicine in very preterm infants.

Cerebral MRI at TEA is a well-established, valuable neuroimaging tool for predicting adverse neurodevelopmental outcomes in preterm infants^11–13,38^. Our study identified the presence of more than one severe BI on cMRI as a key factor strongly associated with cognitive and motor outcomes. Consistent with prior studies, we demonstrated the significance of both the presence and quantity of (severe) BIs on outcome^12,33,38^, underscoring the crucial role of neuroimaging modalities, such as cMRI at TEA in very preterm infants for clinical and prognostic purposes. Moreover, our findings indicate that not only severe but also moderate and mild BIs significantly contribute to cognitive and motor outcomes in preterm infants. While the impact of high-grade IVH on outcome is well known^33^, the impact of low-grade IVH is still being controversially discussed and often attributed as minor significant^15,39–41^. Nevertheless, early cognitive and visual delays in low-grade IVH have been reported in isolated cases, primarily based on ultrasound^13,14,42^. Our study revealed that not only IVH III° and IVH III°+PVHI were associated with adverse cognitive and motor outcomes, but also IVH II° (in contrast to IVH I°). This underscores the importance of accurately classifying IVH as either I° or II° hemorrhage using cMRI^41^, a task facilitated by the utilization of high-hemosensitive sequences like SWI^27^. Furthermore, our results have highlighted CBH as a risk factor for adverse cognitive outcomes, which is consistent with recent emphasis by Volpe et al.^43,44^. Despite the significant role in motor pathways, CBH, PVHI, and cPVL were not significantly associated with adverse motor outcomes in our cohort, contrary to prior studies^33,38,45^, possibly due to the limited number of cases. Infants with CBH (*n* = 33 with outcome data) scored 81.79 ± 22.76 compared to 88.78 ± 18.77 without CBH and infants affected by PVHI (*n* = 3 with available outcome data) had a motor outcome score of 66.00 ± 19.00, while those not affected by PVHI scored 88.3 ± 19.23. Although there appears to be an impact on motor outcomes, the results did not reach statistical significance (*p* = 0.081) due to the small sample size of PVHI cases (*n* = 3 with available outcome data), as previously noted in other studies by Goeral and Arulkumaran^34,39^. Similarly, infants with cPVL (*n* = 10 with outcome data) scored 76.00 ± 23.11 for motor outcomes, in contrast to a score of 88.44 ± 19.09 for infants without cPVL. We consider that PVHI and cPVL did not achieve significant results due to sample size limitations. Further research is needed to clarify this correlation. Moderate to severe ventricular dilatation, indicative of white matter loss, also significantly contributed to cognitive and motor outcomes in our cohort. Although it is known that preterm infants often experience ventricular dilatation^46–48^, its impact on the outcome of preterm infants remains unclear and lacks comprehensive data^38,47,48^. To summarize, even moderate and mild brain injuries detected on cMRI, such as IVH II°, moderate/severe ventricular dilatation, and CBH, can be regarded as risk factors for unfavorable outcomes.

This study emphasized the significant impact of perinatal and neonatal management on outcomes, which were influenced by well-documented risk factors in the literature (e.g., GA, birthweight, SGA, catecholamine treatment, sepsis, CPAP/ventilation, ROP, BPD, operations, and PDA^49,50^. However, in our study population, NEC, a known risk factor, was not significant, probably due to the low sample size. The Apgar score, as a known risk factor for adverse outcomes,^16^ emerged as a key player for cognitive and motor outcomes. Continuous score values were analyzed to mitigate observer bias and limitations of the score for preterm infants. Surfactant administration emerged as an independent protective key factor for motor outcome, indicating its role in addressing surfactant deficiency and stabilizing respiratory conditions during initial care^51^. It has been demonstrated that surfactant administration increases survival rates among extremely premature infants, but it did not significantly impact outcomes. Considering that previous studies often focused solely on infants with severe adverse outcomes (< 70) to describe the effect of surfactants, our approach accounts for mild to moderate changes by utilizing continuous outcomes score values, which can significantly impact an infant’s life^52^. Clinical trials on transfusions of RBCs revealed mixed results^53–57^. The ETTNO trial (*n* = 1013) found no significant difference in neurodevelopmental outcomes between threshold-related restrictive and liberal transfusion strategies. As an ancillary finding, they indicate a volume-dependent mild effect on neurodevelopmental outcomes^55^. However, we showed that the number of transfusions of RBCs was an independent key risk factor for adverse cognitive and motor outcomes. Moreover, our data revealed a threshold (> five transfusions), beyond which outcomes declined extremely. Comorbidities related to prematurity and anemia of the preterm infant may be the cause, but there are also indications that transfusions themselves may have adverse effects^57^. In conclusion, perinatal and neonatal risk factors, as indicators of the clinical condition of preterm infants, significantly influence cognitive and motor outcomes, with APGAR score, surfactant administration, and transfusion of RBCs emerging as key factors in our cohort.

Our study has some limitations that need to be considered. Firstly, we conducted an exploratory analysis without adjustment for multiple comparisons, utilizing data from a retrospective single-center design. Consequently, the associations observed in our data should be interpreted cautiously and require validation in future studies. We would like to acknowledge that this study utilized cMRI at TEA as the neuroimaging modality and did not include cranial ultrasound findings as a comparison of neuroimaging methods is beyond the scope of this study. Future studies comparing these two modalities are necessary. Approximately one-third of the infants were lost to follow-up, declined testing, leading to potential selection biases. Additionally, due to the ten-year examination timeframe and retrospective design, different editions of the Bayley Scales of Infant Development (BSID II/III) were employed. However, Fuiko et al. reported that German norms did not overestimate performance compared to American norms, suggesting comparability between edition^58^. As a robustness check, we controlled for the different BSID editions in our regression analyses and did not find any significant associations between the BSID editions and the cognitive outcome. We observed missing cognitive outcome data in 165/507 and missing motor outcome data in 201/501 infants. A higher proportion of loss to follow-up was found in the group of more mature infants. This may be attributed to the fact that parents of these infants do not attend follow-up appointments so regularly, as they are satisfied with their infant’s development and do not perceive the need for continued follow-up as a priority, compared to the parents of extremely immature preterm infants. However, we do not find any significant differences in missing outcome data with regards to brain injuries with clinical relevance.

In conclusion, we identified risk factors for adverse neurodevelopmental outcomes of very preterm infants encompassing cMRI-detected brain injuries and clinical factors. Even mild brain injuries such as IVH II° and moderate ventricular dilatation significantly impacted neurological outcomes in very preterm infants. We observed that a composite consideration of these factors might aid in outcome prediction, elucidating the most influential key factors on cognitive and motor outcomes. These findings offer valuable insights for risk stratification and outcome prediction in high-risk preterm infants and may serve as useful tools for clinical practice in managing patients and structures postnatal care planning.

**Table 1:** Association between a) motor outcome (MO) and perinatal factors, b) cognitive outcome (CO) and perinatal factors.

a) Association between motor outcome (MO) and perinatal factors				
Perinatal factors	*n*/*N* (%)	MO median (25–75%)	MO mean ± SD	*p*-value
Weeks of gestation at birth (weeks)				< 0.001
23–25	55 (18)	78 (58–89)	76.8 ± 21.09	
26–28	102 (33.3)	89 (73–100)	87.01 ± 19.43	
29–31	149 (48.7)	95 (83–106)	92.87 ± 16.67	
Birthweight (g)				< 0.001
≤500	12 (3.9)	61 (50–76)	63.08 ± 13.10	
501–1000	114 (37.3)	85 (72–96)	84.76 ± 20.11	
1001–1500	132 (43.1)	92 (80–100)	89.41 ± 16.72	
>1500	48 (15.7)	101.5 (87–113)	98.23 ± 18.27	
Birthweight < 1000 g				< 0.001
Yes	126 (41.2)	82 (69–96)	82.7 ± 20.53	
No	180 (58.8)	95 (82–106)	91.76 ± 17.53	
Percentile (%)				0.010
≤25	127(41.9)	85 (77–96)	85.28 ± 17.96	
25–50	97 (32.0)	88 (76–100)	87.06 ± 19.97	
50–75	65 (21.5)	96 (82–107)	94.43 ± 18.44	
>75	14 (4.6)	97.5 (64–109)	89.64 ± 24.78	
Sex				0.358
Female	158 (51.6)	90 (77–103)	88.67 ± 20.15	
Male	148 (48.4)	88.5 (76.5–100)	87.34 ± 18.42	
Delivery				0.111
Vaginal	13 (4.3)	96 (85–109)	95.46 ± 18.68	
Primary cesarean section	226 (73.9)	88.5 (77–100)	86.89 ± 18.94	
Secondary cesarean section	67 (21.9)	89 (76–106)	90.42 ± 20.43	
Multiple births				0.294
Yes	92 (30.1)	90.5 (79–104.5)	90.29 ± 18.33	
No	214 (69.9)	89 (76–100)	87.06 ± 19.69	
PPROM (h)				0.140
0	196 (73.7)	88.5 (76.5–103)	87.59 ± 19.45	
0–18	14 (5.3)	88 (77–96)	83.21 ± 20.48	
18–168 (1 week)	36 (13.5)	92 (87–106)	92.56 ± 17.28	
>168 (> 1 week)	20 (7.5)	83 (67–98)	80.55 ± 20.48	
SGA				0.025
Yes	43 (14.2)	84 (72–96)	82.02 ± 18.66	
No	260 (85.8)	90 (79–103)	89.00 ± 19.25	
Admission temperature (C°)				0.251
<36 °C	17 (5.9)	80 (78–103)	85.06 ± 20.15	
36–37.5 °C	236 (81.4)	89 (78.5–103)	88.59 ± 19.38	
≥37.6 °C	37 (12.8)	84 (72–96)	83.73 ± 20.01	
Emergency cesarean section				0.249
Yes	38 (12.4)	86.5 (68–100)	84.21 ± 19.70	
No	268 (87.6)	89 (79–101.5)	88.57 ± 19.24	
Apgar score 1/5/10 minutes				
1 min				0.009
0–5 score points	71 (23.3)	82 (61–103)	82.45 ± 22.90	
6–10 score points	234 (76.7)	92 (79–100)	89.74 ± 17.84	
5 min				0.074
0–5 score points	7 (2.3)	78 (58–84)	76.00 ± 16.58	
6–10 score points	299 (97.7)	89 (77–103)	88.31 ± 19.31	
10 min				0.615
0–5 score points	2 (0.7)	76 (49–103)	76.00 ± 38.18	
6–10 score points	304 (99.4)	89 (77–100)	88.11 ± 19.23	
Surfactant				0.043
Yes	150 (50.8)	86.5 (72–100)	85.82 ± 20.71	
No	155 (49.2)	92 (80–103)	90.41 ± 17.44	
Catecholamine treatment				< 0.001
Yes	21 (6.9)	70 (50–84)	70.19 ± 20.86	
No	285 (93.1)	92 (79–103)	89.34 ± 18.57	

b) Association between cognitive outcome (CO) and perinatal factors				
Perinatal factors	–*n*/*N* (%)	CO median (25–75%)	CO mean ± SD	*p*-value
Weeks of gestation at birth (weeks)				< 0.001
23–25	63 (18.4)	85 (75–98)	84.79 ± 18.35	
26–28	114 (33.3)	95 (80–106)	91.77 ± 20.16	
29–31	165 (48.3)	99 (85–110)	96.94 ± 18.13	
Birthweight (g)				< 0.001
≤500	12 (3.5)	72.5 (50–85.25)	71.21 ± 18.94	
501–1000	133 (38.9)	90 (80–105)	91.32 ± 18.99	
1001–1500	141 (41.2)	98 (85–109)	94.90 ± 19.35	
>1500	56 (16.4)	98 (85–110)	96.75 ± 17.27	
Birthweight < 1000 g				0.005
Yes	145 (42.4)	90 (78–104)	89.66 ± 19.72	
No	197 (57.6)	98 (85–110)	95.42 ± 18.75	
Percentile (%)				0.441
≤25	139 (41.0)	92 (80–105)	90.91 ± 19.49	
25–50	112 (33.0)	97.5 (81–110)	94.32 ± 20.09	
50–75	71 (20.9)	95 (85–110)	95.58 ± 18.41	
>75	17 (5.0)	95 (85–105)	91.82 ± 16.90	
Sex				0.687
Female	174 (50.9)	94 (80–109)	92.70 ± 19.75	
Male	168 (49.1)	95 (82–105)	93.26 ± 18.99	
Delivery				0.381
Vaginal	14 (4.1)	94 (85–111)	96.43 ± 20.36	
Primary Cesarean section	256 (74.9)	95 (82–109.5)	93.62 ± 19.61	
Secondary Cesarean section	72 (21.1)	93 (75–105)	90.05 ± 18.13	
Multiple births				0.314
Yes	102 (29.8)	95 (84–108)	94.74 ± 18.22	
No	240 (70.2)	94 (80–106)	92.23 ± 19.80	
PPROM (h)				0.072
0	218 (72.9)	94 (80–106)	92.75 ± 19.67	
0–18	17 (5.7)	99 (90–102)	93.82 ± 17.29	
18–168 (1 week)	42 (14.1)	95 (88–110)	97.01 ± 16.62	
>168 (> 1 week)	22 (7.4)	81 (70–101)	82.59 ± 19.97	
SGA				0.208
Yes	51 (15.0)	90 (75–105)	89.47 ± 20.74	
No	288 (85.0)	95 (84–108.5)	93.69 ± 19.08	
Admission temperature (°C)				0.319
≤36 °C	20 (6.1)	90.5 (84.5–104)	91.70 ± 16.00	
36–37.5 °C	262 (80.4)	95 (84–110)	93.86 ± 19.54	
≥37.6 °C	44 (13.5)	94.5 (75–100)	88.82 ± 20.58	
Emergency cesarean section				0.351
Yes	43 (12.6)	90 (80–105)	91.51 ± 18.89	
No	299 (87.4)	95 (82–108)	93.19 ± 19.44	
Apgar score 1/5/10 minutes				
1 min				0.027
0–5 score points	81 (23.8)	90 (75–102)	88.52 ± 21.6	
6–10 score points	260 (76.3)	95 (84–109)	94.38 ± 18.46	
5 min				0.026
0–5 score points	9 (2.6)	85 (75–90)	80.56 ± 13.49	
6–10 score points	333 (97.4)	95 (82–108)	93.32 ± 19.39	
10 min				0.153
0–5 score points	2 (0.6)	72.5 (60–85)	72.50 ± 17.68	
6–10 score points	340 (99.4)	95 (81.5–107.5)	93.10 ± 19.32	
Surfactant				0.105
Yes	169 (49.6)	92 (80–105)	91.10 ± 19.98	
No	172 (50.4)	95.5 (84–110)	94.85 ± 18.64	
Catecholamine treatment				< 0.001
Yes	23 (6.7)	75 (55–94)	75.96 ± 21.86	
No	319 (93.3)	95 (84–109)	94.21 ± 18.60	

*CO*: cognitive outcome, *MO*: motor outcome, *PPROM*: preterm premature rupture of membranes, *SGA*: small for gestational age, significant: *p* < 0.05.

**Table 2:** Association between a) motor outcome (MO) and neonatal factors, b) cognitive outcome (CO) and neonatal factors.

a) Association between motor outcome (MO) and neonatal factors				
Neonatal factors	*n*/*N* (%)	MO median (25–75%)	MO mean ± SD	*p*-value
Sepsis				< 0.001
Yes	99 (32.4)	82 (67–96)	81.47 ± 20.24	
No	207 (67.7)	93 (80–103)	91.16 ± 18.08	
CPAP (d)				0.002
<20	146 (48.2)	96 (80–106)	91.91 ± 18.08	
20–39	86 (28.4)	85 (76–100)	86.38 ± 18.40	
40–59	56 (18.5)	86 (69–100)	84.57 ± 21.53	
≥60	15 (5.0)	82 (50–93)	74.87 ± 18.61	
ROP				< 0.001
Yes	106 (35.2)	82 (67–94)	80.50 ± 18.73	
No	195 (64.8)	95 (82–106)	91.87 ± 18.51	
Transfusion of red blood cells				< 0.001
0	185 (60.5)	96 (84–106)	93.60 ± 16.53	
1–5	104 (34.0)	82 (72–96)	83.03 ± 18.96	
6–10	12 (3.9)	50 (50–55.5)	55.50 ± 10.77	
>10	6 (1.6)	53 (50–82)	64.00 ± 17.87	
NEC				0.289
No	298 (97.4)	89 (78–103)	88.34 ± 19.15	
No surgery	5 (1.6)	79 (76–88)	81.60 ± 22.03	
surgery	3 (1.0)	54 (50–100)	68.00 ± 27.78	
BPD (36 weeks of GA)				< 0.001
Yes	45 (14.7)	78 (57–84)	73.31 ± 17.20	
No	261 (85.3)	92 (80–103)	90.57 ± 18.54	
surgery				< 0.001
Yes	79 (25.8)	82 (61–96)	79.94 ± 20.51	
No	227 (74.2)	92 (80–106)	90.85 ± 18.10	
Ventilation (invasive, d)				< 0.001
<10	261 (85.3)	92 (81–103)	91.38 ± 17.49	
10–19	24 (7.8)	70 (52–80)	70.46 ± 17.70	
20–29	10 (3.3)	50 (50–61)	59.80 ± 16.88	
30–39	4 (1.3)	74 (62.5–86)	74.25 ± 17.63	
≥40	7 (2.3)	82 (50–85)	71.57 ± 21.01	
PDA				< 0.001
No PDA	54 (17.7)	89 (79–103)	88.59 ± 19.87	
PDA: no treatment	109 (35.7)	96 (82–106)	92.68 ± 16.61	
PDA: pharmacological treatment	127 (41.6)	88 (73–100)	86.46 ± 19.77	
PDA: intervention	15 (4.9)	65 (50–78)	65.67 ± 16.10	
PDA				0.797
Yes	251 (82.3)	89 (77–100)	87.92 ± 19.27	
No	54 (17.7)	89 (79–103)	88.59 ± 19.87	

b) Association between cognitive outcome (CO) and neonatal factors				
Neonatal factors	*n*/*N* (%)	CO median (25–75%)	CO mean ± SD	*p*-value
Sepsis				0.022
Yes	109 (31.9)	90 (80–102)	89.28 ± 19.44	
No	233 (68.1)	95 (84–109)	94.71 ± 19.11	
CPAP (d)				0.002
<20	166 (49.0)	98 (85–110)	95.89 ± 18.39	
20–39	94 (27.7)	93 (78–103)	91.10 ± 19.86	
40–59	63 (18.6)	95 (82–110)	92.73 ± 19.74	
≥60	16 (4.7)	80 (67.5–87.5)	77.88 ± 16.82	
ROP				< 0.001
Yes	122 (36.3)	88 (75–102)	87.15 ± 18.90	
No	214 (63.7)	98 (85–110)	96.10 ± 19.00	
Transfusion of red blood cells				< 0.001
0	205 (59.9)	99 (85–110)	96.84 ± 17.49	
1–5	118 (34.5)	90.5 (80–105)	90.51 ± 19.37	
6–10	14 (4.1)	65.5 (50–80)	65.68 ± 15.1	
>10	5 (1.5)	75 (50–78)	69.40 ± 19.13	
NEC				0.278
No	332 (97.1)	95 (82–107.5)	93.25 ± 19.10	
No surgery	6 (1.8)	87 (80.5–114)	90.58 ± 26.51	
surgery	4 (1.2)	72.5 (52.5–95)	73.75 ± 24.96	
BPD (36 weeks of GA)				< 0.001
Yes	52 (15.2)	86 (68–92)	82.53 ± 18.43	
No	290 (84.8)	96 (84–110)	94.85 ± 18.94	
Surgery				< 0.001
Yes	93 (27.2)	89 (72–100)	85.55 ± 21.11	
No	249 (72.8)	95 (85–110)	95.75 ± 17.92	
Ventilation (invasive, d)				< 0.001
<10	290 (84.8)	98 (85–110)	96.07 ± 17.95	
10–19	27 (7.9)	82 (62–90)	76.59 ± 17.76	
20–29	12 (3.5)	72.5 (52.5–87.5)	72.67 ± 18.39	
30–39	5 (1.5)	80 (78–80.5)	83.90 ± 19.03	
≥40	8 (2.3)	70.5 (57–87)	72.38 ± 18.55	
PDA				< 0.001
No PDA	58 (17.0)	92.5 (80–103)	91.65 ± 17.82	
PDA: no treatment	125 (36.7)	100 (85–110)	96.53 ± 18.75	
PDA: pharmacological treatment	141 (41.4)	94 (84–105)	92.45 ± 19.49	
PDA: intervention	17 (5.0)	80 (62–88)	75.65 ± 19.37	
PDA				0.309
Yes	283 (83.0)	95 (82–109)	93.24 ± 19.7	
No	58 (17.0)	92.5 (80–103)	91.65 ± 17.82	

*CO*: cognitive outcome, *CPAP*: continuous positive airway pressure, *BPD*: bronchopulmonary dysplasia, *MO*: motor outcome, *NEC*: necrotizing enterocolitis, *PDA*: patent ductus arteriosus, *ROP*: retinopathy of prematurity, significant: *p* < 0.05.

**Table 3:** Association between a) motor outcome (MO) and cMRI- detected brain injuries, b) cognitive outcome (CO) and cMRI- detected brain injuries.

a) Association between motor outcome (MO) and cMRI- detected brain injuries				
Brain injuries	*n*/*N* (%)	MO median (25–75%)	MO mean ± SD	*p*-value
IVH				< 0.001
None	252 (82.4)	92 (79–103)	89.73 ± 17.91	
IVH I°	21 (6.9)	100 (78–110)	91.48 ± 26.45	
IVH II°	24 (7.8)	73 (57–88.5)	74.79 ± 19.19	
IVH III°	9 (2.9)	67 (50–77)	67.56 ± 15.36	
IVH III ° + PVHI				< 0.001
Yes	10 (3.3)	66 (50–77)	66.90 ± 14.63	
No	296 (96.7)	90 (79–103)	88.74 ± 19.07	
IVH I°				0.430
Bilateral	7 (33.3)	103 (78–125)	98.86 ± 29.44	
Unilateral	14 (66.7)	96 (58–106)	87.79 ± 25.15	
PVHI				0.081
Yes	3 (1.0)	61 (50–87)	66.00 ± 19.00	
No	303 (99.0)	89 (77–103)	88.3 ± 19.23	
Ventricular dilatation				0.002
None	26 (8.5)	90.5 (82–106)	92.85 ± 16.69	
Mild	204 (66.7)	92 (79–103)	90.21 ± 19.04	
Moderate	64 (20.9)	84.5 (68.5–95.5)	81.61 ± 18.58	
Severe	12 (3.9)	75 (50–94.5)	74.75 ± 21.65	
CBH				0.093
Yes	33 (10.8)	82 (61–100)	81.79 ± 22.76	
No	273 (89.2)	89 (79–103)	88.78 ± 18.77	
CBH Score (Kidokoro)				0.305
None	273 (89.2)	89 (79–103)	88.78 ± 18.77	
CBH I°	14 (4.6)	82 (72–96)	83.21 ± 22.03	
CBH II°	3 (1.0)	100 (61–113)	91.33 ± 27.06	
CBH III°	7 (2.3)	76 (50–100)	73.00 ± 23.47	
CBH IV°	9 (2.9)	85 (70–96)	83.22 ± 24.07	
cPVL				0.096
Yes	10 (3.3)	74.5 (50–100)	76.00 ± 23.11	
No	296 (96.7)	89 (78.5–101.5)	88.44 ± 19.09	
Punctate white matter lesions				0.338
No	247 (80.7)	89 (79–103)	88.77 ± 18.40	
Bilateral	42 (13.7)	90.5 (73–103)	87.00 ± 22.19	
Unilateral	17 (5.6)	80 (60–98)	79.82 ± 23.77	
≥6				0.829
Yes	26 (8.5)	90.5 (76–106)	88.19 ± 21.90	
No	280 (91.5)	89 (77.5–100)	88.01 ± 19.10	
≥10				0.105
Yes	10 (3.3)	77.5 (67–89)	78.60 ± 20.82	
No	296 (96.7)	89 (78–103)	88.35 ± 19.22	
DEHSI				0.364
Yes	184 (98.4)	89 (77–100)	87.85 ± 19.40	
No	3 (1.6)	92 (82–106)	95.57 ± 14.29	
Number of brain injuries				< 0.001
≤0	147 (48.0)	92 (80–103)	90.63 ± 17.32	
1–2	141 (46.1)	89 (77–100)	87.68 ± 19.76	
≥3	18 (5.9)	64 (50–82)	69.56 ± 21.94	
> 1 brain injury				< 0.001
Yes	56 (18.3)	78 (54–92)	76.63 ± 21.02	
No	250 (81.7)	92 (80–103)	90.58 ± 17.99	
Severe brain injuries				0.047
Yes	39 (12.8)	82 (65–100)	81.23 ± 21.22	
No	267 (87.3)	89 (79–103)	89.02 ± 18.86	
Number of severe brain injuries				0.035
≤0	267 (87.3)	89 (79–103)	89.02 ± 18.86	
1–2	35 (11.4)	85 (67–100)	83.09 ± 20.96	
≥3	4 (1.3)	61.5 (50–80)	65.00 ± 18.24	
> 1 severe brain injury				< 0.001
Yes	10 (3.3)	63 (50–76)	63.90 ± 13.81	
No	296 (96.7)	90 (79–103)	88.84 ± 18.96	

b) Association between cognitive outcome (CO) and cMRI- detected brain injuries				
Brain injuries	–*n*/*N* (%)	CO median (25–75%)	CO mean ± SD	*p*-value
IVH				0.002
None	281 (82.2)	95 (84–108)	94.31 ± 18.67	
IVH I°	22 (6.4)	100 (80–114)	94.95 ± 20.70	
IVH II°	27 (7.9)	85 (75–93)	85.02 ± 20.81	
IVH III°	12 (3.5)	77.5 (58.5–87)	76.17 ± 19.37	
IVH III ° +PVHI				0.002
Yes	13 (3.8)	75 (62–86)	76.08 ± 18.55	
No	329 (96.2)	95 (82–108)	93.65 ± 19.11	
IVH I°				0.456
Bilateral	7 (31.8)	100 (92–115)	102.00 ± 17.00	
Unilateral	15 (68.2)	100 (75–110)	91.67 ± 21.96	
PVHI				0.390
Yes	3 (0.9)	80 (75–101)	85.33 ± 13.80	
No	339 (99.1)	95 (82–108)	93.05 ± 19.40	
Ventricular dilatation				0.004
None	27 (7.9)	98 (85–105)	94.63 ± 15.33	
Mild	227 (66.4)	95 (85–110)	95.20 ± 18.44	
Moderate	72 (21.1)	86.5 (75–102.5)	88.15 ± 21.31	
Severe	16 (4.7)	85 (58.5–99.5)	80.41 ± 21.73	
CBH				0.029
Yes	36 (10.5)	85 (70–100)	85.57 ± 21.75	
No	306 (89.5)	95 (84–108)	93.85 ± 18.90	
CBH Score (Kidokoro)				0.273
None	306 (89.5)	95 (84–108)	93.85 ± 18.90	
CBH I°	16 (4.7)	89 (65.5–99.5)	85.44 ± 23.78	
CBH II°	3 (0.9)	80 (75–115)	90.00 ± 21.79	
CBH III°	8 (2.3)	82.5 (75–94)	84.88 ± 14.61	
CBH IV°	9 (2.6)	80.5 (70–111)	84.94 ± 26.35	
cPVL				0.076
Yes	13 (3.8)	80.5 (75–94)	84.35 ± 19.40	
No	329 (96.2)	95 (82–108)	93.32 ± 19.30	
Punctate white matter lesions				0.503
No	274 (80.1)	95 (82–106)	93.36 ± 18.86	
Bilateral	50 (14.6)	96.5 (84–110)	93.42 ± 20.10	
Unilateral	18 (5.3)	87.5 (66–107)	85.89 ± 24.11	
≥6				0.799
Yes	31 (9.1)	98 (82–110)	92.58 ± 21.30	
No	311 (90.9)	95 (80.5–106)	93.02 ± 19.18	
≥10				0.447
Yes	13 (3.8)	87 (60–105)	86.54 ± 24.25	
No	329 (96.2)	95 (82– 107)	93.23 ± 19.14	
DEHSI				0.289
Yes	184 (98.4)	95 (80.5–106.5)	92.75 ± 19.21	
No	3 (1.6)	100 (84–120)	104.14 ± 24.19	
Number of brain injuries				< 0.001
0	165 (48.3)	95 (85–106)	94.90 ± 17.27	
1–2	155 (45.3)	95 (80–110)	93.38 ± 20.02	
≥3	22 (6.4)	75.5 (55–88)	75.70 ± 21.67	
> 1 brain injury				< 0.001
Yes	67 (19.6)	85 (70–97)	83.43 ± 19.99	
No	275 (80.4)	96 (85–110)	95.31 ± 18.50	
Severe brain injuries				0.007
Yes	46 (13.5)	85 (70–101)	85.22 ± 20.45	
No	296 (86.6)	95 (84–108)	94.19 ± 18.93	
Number of severe brain injuries				0.017
0	296 (86.6)	95 (84–108)	94.19 ± 18.93	
1–2	41 (12.0)	86 (70–105)	86.15 ± 20.67	
≥3	5 (1.5)	80 (72–85)	77.60 ± 18.72	
> 1 severe brain injury				< 0.001
Yes	13 (3.8)	75 (55–85)	71.88 ± 16.95	
No	329 (96.2)	95 (84–108)	93.81 ± 18.99	

*Brain injury*: IVH I°-III°, PVHI, moderate and severe VD, CBH, punctate white matter lesions, cPVL, *CBH*: cerebellar hemorrhage, *CO*: cognitive outcome, *cPVL*: cystic periventricular leukomalacia, *DEHSI*: diffuse excessive high signal intensity, *IVH*: intraventricular hemorrhage, *MO*: motor outcome, *PVHI*: periventricular hemorrhagic infarction, *severe brain injury*: IVH III°, PVHI, CBH III°+IV°, severe VD, cPVL, significant: *p* < 0.05.

**Table 4:** a) Motor outcome (MO) - Linear regression, b) Cognitive outcome (CO) - Linear Regression

a) Motor outcome (MO) - Linear Regression																									
	Univariable Analysis					Model 1 (perinatal factors)					Model 2 (neonatal factors)					Model 3 (brain injuries)					Model 4 (key factors)				
						Adj. *R*-squared: 0.199					Adj. *R*-squared: 0.187					Adj. *R*-squared: 0.087					Adj. *R*-squared: 0.233				
	Coef.	CI95		*P* value		Coef.	CI95		*P* value		Coef.	CI95		*P* value		Coef.	CI95		*P* value		Coef.	CI95		*P* value	
Days birth to outcome measurement – (adjusted)	-0.00	-0.02–0.02		0.941																					
Perinatal factors																									
Weeks of gestation at birth –weeks	2.63	1.69–3.56		< 0.001		2.72	1.17–4.27		0.001												0.73	-0.56–2.03		0.265	
Birthweight < 1000 g	-9.06	-13.48 – -4.64		< 0.001		3.78	-3.08–10.64		0.279																
Percentile (%)	0.15	0.06–0.25		0.002																					
SGA	-6.98	-13.01 – -0.95		0.023		-8.34	-14.23 – -2.45		0.006												-4.47	-9.84–0.89		0.102	
Apgar score 1/5/10 minutes (continuous score values)																									
10 min	6.48	4.10–8.86		< 0.001		5.61	3.42–7.81		< 0.001												5.30	3.31–7.30		< 0.001	
Surfactant	-4.59	-8.91 – -0.27		0.037		5.53	1.02–10.03		0.016												4.88	0.44–9.32		0.031	
Catecholamine treatment	-19.15	-28.19 – -10.12		< 0.001		-14.45	-23.49 – -5.42		0.002												-6.73	-16.42–2.96		0.172	
Neonatal factors																									
Sepsis	-9.69	-14.39 – -4.99		< 0.001							-2.40	-7.21–2.40		0.325											
CPAP (days)	-0.20	-0.31 – -0.09		< 0.001							0.01	-0.11–0.13		0.848											
ROP	-11.37	-15.79 – -6.94		< 0.001							-5.07	-9.85 – -0.28		0.038							-2.92	-7.60–1.76		0.220	
Transfusion of RBCs (continuous score values)	-3.19	-4.22 – -2.16		< 0.001							-1.73	-3.33 – -0.13		0.034							-1.69	-2.89 – -0.49		0.006	
BPD	-17.26	-22.75 – -11.76		< 0.001							-6.48	-13.69–0.73		0.078											
surgery	-10.91	-16.02 – -5.80		< 0.001																					
Ventilation (invasive) (d)	-0.77	-1.07 – -0.47		< 0.001							-0.12	-0.59–0.35		0.618											
PDA intervention (Yes/No)	-23.53	-31.75 – -15.31		< 0.001							-2.20	-12.40–8.00		0.672											
Brain injuries																									
IVH II°	-14.94	-22.86 - -7.03		< 0.001																					
IVH III°	-22.18	-32.00- -12.36		< 0.001																					
IVH III °+PVHI	-21.84	-30.78 – -12.91		< 0.001												-0.87	-15.69–13.94		0.908						
PVHI	-22.25	-40.06- -4.43		0.015																					
Ventricular dilatation (VD)																									
None	Ref															Ref									
Mild	-2.64	-9.52–4.25		0.452												-1.99	-8.91–4.93		0.572						
Moderate	-11.24	-19.07 – -3.41		0.005												-6.16	-14.22–1.90		0.133						
Severe	-18.10	-31.55 – -4.64		0.009												2.48	-7.37–12.33		0.621						
Ventricular dilatation moderate/severe	-9.98	-14.93- -5.04		< 0.001																					
Number of brain injuries																									
>1 brain injury	-13.96	-19.90 – -8.02		< 0.001												-8.80	-15.95 – -1.66		0.016						
Number of severe brain injuries																									
>1 severe brain injury	-24.94	-33.41 – -16.48		< 0.001												-20.19	-37.35 – -3.04		0.021		-11.27	-20.84 – -1.70		0.021	

b) Cognitive outcome (CO) - Linear Regression																									
	Univariable Analysis					Model 1 (perinatal factors)					Model 2 (neonatal factors)					Model 3 (brain injuries)					Model 4 (key factors)				
						Adj. *R*-squared: 0.128					Adj. *R*-squared: 0.129					Adj. *R*-squared: 0.068					Adj. *R*-squared: 0.178				
	Coef.		CI95		*P* value	Coef.		CI95		*P* value	Coef.		CI95		*P* value	Coef.		CI95		*P* value	Coef.		CI95		*P* value
Days birth to outcome measurement (adjusted)	0.01		-0.01–0.03		0.487																				
Perinatal factors																									
Weeks of gestation at birth –weeks	1.91		1.04–2.78		< 0.001	1.18		-0.31–2.68		0.119															
Birthweight < 1000 g	-5.77		-9.92– -1.61		0.007	0.99		-5.60–7.58		0.768															
Apgar score 1/5/10 minutes (continuous score values)																									
10 min	5.71		3.44–7.99		< 0.001	4.90		2.68–7.12		< 0.001											4.45		2.55–6.35		< 0.001
Catecholamine treatment	-18.25		-27.28 – -9.22		< 0.001	-15.90		-25.08 – -6.72		0.001											-6.95		-16.73–2.82		0.163
Neonatal factors																									
Sepsis	-5.43		-9.84 – -1.02		0.016						1.49		-2.94–5.92		0.509										
CPAP (days)	-0.19		-0.28 – -0.09		< 0.001						-0.04		-0.14–0.07		0.457										
ROP	-8.95		-13.17 – -4.73		< 0.001						-3.14		-7.70–1.41		0.176										
Transfusion of RBCs (continuous score values)	-2.89		-3.85 – -1.93		< 0.001						-1.87		-3.33 – -0.41		0.012						-1.96		-3.04 – -0.88		< 0.001
BPD	-12.32		-17.78 – -6.87		< 0.001						-1.79		-8.53–4.95		0.602										
surgery	-10.20		-15.04 – -5.36		< 0.001																				
Ventilation (invasive) (days)	-0.62		-0.88 – -0.36		< 0.001						-0.19		-0.52–0.15		0.269										
PDA intervention (Yes/No)	-18.23		-27.46 – -9.01		< 0.001						-0.24		-10.79–10.31		0.965										
Brain injuries																									
IVH II°	-9.29		-17.3 – -1.21		0.024																				
IVH III°	-18.14		-28.96 – -7.32		0.001																				
IVH III °+PVHI	-17.57		-27.54 – -7.60		0.001											6.37		-11.42–24.16		0.482					
VD	7.71		-5.28 – -20.71																						
Severe	-14.22		-26.10 – -2.34		0.019																				
VD moderate/severe	-8.40		-13.42 – -3.39		0.001											-3.13		-9.01–2.75		0.296					
CBH (Yes/No)	-8.28		-15.65 – -0.92		0.028											-2.53		-10.28–5.21		0.520					
Number of brain injuries																									
>1 brain injury	-11.88		-17.14 – -6.62		< 0.001											-6.92		-13.99–0.15		0.055					
Severe brain injuries	-8.97		-15.24 – -2.70		0.005																				
>1 severe brain injury	-21.93		-31.07 – -12.78		< 0.001											-18.35		-35.91 – -0.78		0.041	-10.30		-20.26 – -0.33		0.043

*Brain injury*: IVHI°-III°, PVHI, moderate and severe VD, CBH, punctate white matter lesions, cPVL, *BPD*: bronchopulmonary dysplasia, *CBH*: cerebellar hemorrhage, *CO*: cognitive outcome, *CPAP*: continuous positive airway pressure, *cPVL*: cystic periventricular leukomalacia, *DEHSI*: diffuse excessive high signal intensity, *IVH*: intraventricular hemorrhage, *MO*: motor outcome, *NDO*: neurodevelopmental outcome, *NEC*: necrotizing enterocolitis, *PDA*: patent ductus arteriosus, *PPROM*: preterm premature rupture of membranes, *PVHI*: periventricular hemorrhagic infarction, *ROP*: retinopathy of prematurity, severe brain injury: IVH III°, PVHI, CBH III°+IV°, severe VD, cPVL, *SGA*: small for gestational age, *Transfusion of RBCs*: transfusion of red blood cells, *VD*: ventricular dilatation (mild, moderate, severe), significant p:<0.05.

## Electronic supplementary material

Below is the link to the electronic supplementary material.

Supplementary Material 1

## Data Availability

The dataset used and/or analysed for the study is available from the corresponding author upon reasonable request.
