# Supplementary material for: CMRI-detected brain injuries and clinical key risk factors associated with adverse neurodevelopmental outcomes in very preterm infants
Source: Sci Rep. 2025 May 25;15:18221. doi: 10.1038/s41598-025-02539-1 (PMC12104413; doi:10.1038/s41598-025-02539-1)
Supplement: Supplementary file 1 — Supplementary Material 1 [file 41598_2025_2539_MOESM1_ESM.pdf]

CMRI-detected brain injuries and clinical key risk factors associated with adverse neurodevelopmental outcomes in very preterm infants

Karla Drommelschmidt* [1,2], Thomas Mayrhofer [3], Hanna Müller [5], Borek Foldyna[4], Janika Raudzus[3], Sophia L. Göricke[6], Bernd Schweiger[6], Selma Sirin[7]

[1]Department of Pediatrics I, Neonatology, Pediatric Intensive Care, and Pediatric Neurology, University Hospital Essen, University of Duisburg-Essen, Essen, Germany

[2]Center for Translational Neuro- and Behavioral Sciences (cTNBS), University Hospital Essen, University of Duisburg-Essen, Essen, Germany

[3] School of Business Studies, Stralsund University of Applied Sciences, Stralsund, Germany

[4]Cardiovascular Imaging Research Center, Department of Radiology, Massachusetts General Hospital – Harvard Medical School, Boston, USA

[5]Division of Neonatology and Department of Pediatrics, University Hospital of Tuebingen, Tuebingen, Germany

[6]Department of Diagnostic and Interventional Radiology and Neuroradiology, University Hospital Essen, University of Duisburg-Essen, Essen, Germany

[7]Department of Diagnostic Imaging, University Children's Hospital Zurich, University of Zurich, Zürich, Switzerland

Brief title: Impact of cMRI-detected brain injuries on neurodevelopmental outcomes

Address for correspondence:

Dr. med. Karla Drommelschmidt,
Department of Pediatrics I
Neonatology, Pediatric Intensive Care, and Pediatric Neurology, University Hospital Essen
University of Duisburg-Essen
Hufelandstraße 55
45147 Essen, Germany

Phone: +49/1776757604, E-mail: Karla.Drommelschmidt@uk-essen.de

Supplemental Table 1

Motor outcome (MO) - Linear Regression

	**Univariable Analysis**			**Model 1 (perinatal factors)**			**Model 2 (neonatal factors)**			**Model 3 (brain injuries)**			**Model 4 (key factors)**		
	Coef.	CI95	P value	Coef.	CI95	P value	Coef.	CI95	P value	Coef.	CI95	P value	Coef.	CI95	P value
**Days birth to outcome measurement** – (adjusted)	-0.00	-0.02 – 0.02	0.941												
**Perinatal factors**															
Weeks of gestation at birth –weeks	2.63	1.69 – 3.56	<0.001	2.72	1.17 – 4.27	0.001							0.73	-0.56 – 2.03	0.265
Birthweight –g	0.02	0.01 – 0.02	<0.001												
Birthweight <1000 g	-9.06	-13.48 – -4.64	<0.001	3.78	-3.08 – 10.64	0.279									
Percentile (%)	0.15	0.06 – 0.25	0.002												
Male sex	-1.33	-5.67 – 3.01	0.548												
Delivery															
Vaginal	ref														
Primary Cesarean section	-8.57	-18.72 – 1.58	0.098												
Secondary Cesarean section	-5.04	-16.04 – 5.95	0.367												
Multiple births	3.24	-1.36 – 7.83	0.167												
PPROM (h)*															
0	Ref.														
0 - 18	-4.37	-15.19 – 6.45	0.427												
18 – 168 (1 week)	4.97	-1.30 – 11.24	0.120												
>168 (1 week)	-7.04	-16.31 – 2.24	0.136												
SGA	-6.98	-13.01 – -0.95	0.023	-8.34	-14.23 – -2.45	0.006							-4.47	-9.84 – 0.89	0.102
Admission temperature – (°C)	-1.38	-5.14 – 2.37	0.469												
Emergency Cesarean section	-4.36	-11.00 – 2.28	0.198												
Apgar score 1/5/10 minutes (continuous score values)															
1 minute	1.85	0.58 – 3.12	0.004												

5 minutes	4.29	2.29 – 6.29	<0.001												
10 minutes	6.48	4.10 – 8.86	<0.001	5.61	3.42 – 7.81	<0.001							5.30	3.31 – 7.30	<0.001
Surfactant	-4.59	-8.91 – -0.27	0.037	5.53	1.02 – 10.03	0.016							4.88	0.44 – 9.32	0.031
Catecholamine treatment	-19.15	-28.19 – -10.12	<0.001	-14.45	-23.49 – -5.42	0.002							-6.73	-16.42 – 2.96	0.172
**Neonatal factors**															
Sepsis	-9.69	-14.39 – -4.99	<0.001				-2.40	-7.21 – 2.40	0.325						
CPAP (days)	-0.20	-0.31 – -0.09	<0.001				0.01	-0.11 – 0.13	0.848						
ROP	-11.37	-15.79 – -6.94	<0.001				-5.07	-9.85 – -0.28	0.038				-2.92	-7.60 – 1.76	0.220
Transfusion of RBCs (continuous score values)	-3.19	-4.22 – -2.16	<0.001				-1.73	-3.33 – -0.13	0.034				-1.69	-2.89 – -0.49	0.006
NEC (surgery)	-20.23	-46.18 – 5.72	0.126												
BPD	-17.26	-22.75 – -11.76	<0.001				-6.48	-13.69 – 0.73	0.078						
surgery	-10.91	-16.02 – -5.80	<0.001												
Ventilation (invasive) (d)	-0.77	-1.07 – -0.47	<0.001				-0.12	-0.59 – 0.35	0.618						
PDA															
No PDA	Ref														
PDA: no treatment	4.09	-2.08 – 10.25	0.193												
PDA: pharmacological treatment	-2.13	-8.46 – 4.21	0.509												
PDA: intervention	-22.93	-32.49 – -13.36	<0.001												
PDA intervention (Yes/No)	-23.53	-31.75 – -15.31	<0.001				-2.20	-12.40 – 8.00	0.672						
PDA	-0.67	-6.48 – 5.13	0.820												
**Brain injuries**															
IVH															
None	Ref														
IVH I°	1.74	-9.64 – 13.12	0.763												
IVH II°	-14.94	-22.86 – -7.03	<0.001												
IVH III°	-22.18	-32.00 – -12.36	<0.001												

IVH III ° + PVHI	-21.84	-30.78 – -12.91	<0.001							-0.87	-15.69 – 13.94	0.908			
PVHI	-22.25	-40.06 – -4.43	0.015												
Ventricular dilatation															
None	Ref									Ref					
Mild	-2.64	-9.52 – 4.25	0.452							-1.99	-8.91 – 4.93	0.572			
Moderate	-11.24	-19.07 – -3.41	0.005							-6.16	-14.22 – 1.90	0.133			
Severe	-18.10	-31.55 – -4.64	0.009							2.48	-7.37 – 12.33	0.621			
Moderate/severe ventricular dilatation	-9.98	-14.93 – -5.04	<0.001												
cPVL	-12.44	-26.30 – 1.42	0.078												
CBH (yes/no)	-7.00	-15.02 – 1.02	0.087												
CBH Score (Kidokoro)															
None	Ref														
CBH I°	-5.57	-17.05 – 5.91	0.340												
CBH II°	2.55	-22.86 – 27.96	0.844												
CBH III°	-15.78	-32.24 – 0.67	0.060												
CBH IV°	-5.56	-20.74 – 9.62	0.471												
Punctate white matter lesions															
≥6	0.18	-8.44 – 8.79	0.968												
≥10	-9.75	-22.28 – 2.78	0.127												
DEHSI	-7.72	-17.84 – 2.40	0.134												
Number of brain injuries															
0	Ref														
1	-0.10	-4.77 – 4.56	0.966												
2	-10.65	-17.63 – -3.68	0.003												
3	-18.13	-30.51 – -5.75	0.004												
4	-27.63	-46.14 – -9.11	0.004												
5	-29.63	-32.46 – -26.79	<0.001												
6	-40.63	-43.46 –	<0.001												

		-37.79													
>1 brain injury	-13.96	-19.90 – -8.02	<0.001							-8.80	-15.95 – -1.66	0.016			
Severe brain injuries	-7.79	-14.79 – -0.79	0.029												
Number of severe brain injuries															
0	Ref														
1	-1.82	-9.46 – 5.82	0.640												
2	-25.86	-34.96 – -16.75	<0.001												
3	-19.02	-36.64 – -1.40	0.034												
5	-39.02	-41.31 – -36.74	<0.001												
>1 severe brain injury	-24.94	-33.41 – -16.48	<0.001							-20.19	-37.35 – -3.04	0.021	-11.27	-20.84 – -1.70	0.021

Cognitive outcome (CO) - Linear Regression

	**Univariable Analysis**			**Model 1 (perinatal factors)**			**Model 2 (neonatal factors)**			**Model 3 (brain injuries)**			**Model 4 (key factors)**		
	Coef.	CI95	P	Coef.	CI95	P	Coef.	CI95	P	Coef.	CI95	P	Coef.	CI95	P
**Days birth to outcome measurement** (adjusted)	0.01	-0.01 – 0.03	0.487												
**Perinatal factors**															
Weeks of gestation at birth –weeks	1.91	1.04 – 2.78	<0.001	1.18	-0.31 – 2.68	0.119									
Birthweight –g	0.01	0.01 – 0.02	<0.001												
Birthweight <1000 g	-5.77	-9.92– -1.61	0.007	0.99	-5.60 – 7.58	0.768									
Percentile (%)	0.08	-0.01 – 0.16	0.093												
Male sex	0.56	-3.56 – 4.68	0.789												
Delivery															
Vaginal	Ref														
Primary cesarean section	-2.81	-13.45 – 7.82	0.603												
Secondary cesarean section	-6.38	-17.55 – 4.79	0.262												
Multiple births	2.50	- 1.84 – 6.85	0.258												
PPROM (h)*															
0	Ref.														
0 – 18	1.08	-7.40 – 9.56	0.803												
18 – 168 (1 week)	4.27	-1.40 – 9.94	0.140												
>168 (1 week)	-10.15	-18.81 – -1.50	0.022												
SGA	-4.22	-10.31 – 1.87	0.173												
Admission temperature (C°)	-2.39	-5.82 – 1.03	0.170												
Emergency cesarean section	-1.68	-7.72 – 4.36	0.585												
Apgar score 1/5/10 minutes (continuous score values)															
1 minute	1.54	0.44 – 2.65	0.006												
5 minutes	3.75	2.15 – 5.35	<0.001												
10 minutes	5.71	3.44 – 7.99	<0.001	4.90	2.68 – 7.12	<0.001							4.45	2.55 – 6.35	<0.001

Surfactant	-3.75	-7.87 – 0.36	0.074												
Catecholamine treatment	-18.25	-27.28 – -9.22	<0.001	-15.90	-25.08 – -6.72	0.001							-6.95	-16.73 – 2.82	0.163
**Neonatal factors**															
Sepsis	-5.43	-9.84 – -1.02	0.016				1.49	-2.94 – 5.92	0.509						
CPAP (days)	-0.19	- 0.28 – - 0.09	<0.001				-0.04	-0.14 – 0.07	0.457						
ROP	-8.95	-13.17 – - 4.73	<0.001				-3.14	-7.70 – 1.41	0.176						
Transfusion of RBCs (continuous score values)	-2.89	-3.85 – -1.93	<0.001				-1.87	-3.33 – - 0.41	0.012				-1.96	-3.04 – - 0.88	<0.001
NEC (surgery)	-19.46	-40.88 – 1.96	0.075												
BPD	-12.32	- 17.78 – -6.87	<0.001				-1.79	-8.53 – 4.95	0.602						
surgery	-10.20	- 15.04 – -5.36	<0.001												
Ventilation (invasive) (days)	-0.62	-0.88 – -0.36	<0.001				-0.19	-0.52 – 0.15	0.269						
PDA															
No PDA	ref														
PDA: no intervention	4.89	-0.77 – 10.54	0.090												
PDA pharmacological treatment	0.80	-4.82 – 6.42	0.779												
PDA: intervention	-16.00	-26.12 – -5.88	0.002												
PDA intervention (Yes/No)	-18.23	-27.46 – -9.01	<0.001				-0.24	-10.79 – 10.31	0.965						
PDA (Yes/No)	1.59	-3.53 – 6.72	0.541												
**Brain injuries**															
IVH															
None	Ref														
IVH I°	0.65	-8.16 – 9.46	0.885												
IVH II°	-9.29	-17.37 – -1.21	0.024												
IVH III°	-18.14	-28.96 – -7.32	0.001												
IVH III °+PVHI	-17.57	-27.54 – -7.60	0.001							6.37	-11.42 – 24.16	0.482			
PVHI	7.71	-5.28 – 20.71	0.244												
Ventricular dilatation															

None	Ref														
Mild	0.57	-5.64 – 6.79	0.856												
Moderate	-6.48	-14.04 – 1.08	0.093												
Severe	-14.22	-26.10 – -2.34	0.019												
Moderate/severe ventricular dilatation	-8.40	-13.42 – -3.39	0.001							-3.13	-9.01 – 2.75	0.296			
cPVL	-8.97	-19.38 – 1.43	0.091												
CBH (Yes/No)	-8.28	-15.65 – -0.92	0.028							-2.53	-10.28 – 5.21	0.520			
CBH Score (Kidokoro)															
None	Ref														
CBH I°	-8.41	-20.02 – 3.19	0.155												
CBH II°	-3.85	-24.32 – 16.62	0.712												
CBH III°	-8.98	-18.79 – 0.84	0.073												
CBH IV°	-8.91	-25.46 – 7.64	0.291												
Punctate white matter lesions															
≥6	-0.44	-8.17 – 7.29	0.911												
≥10	-6.70	-19.61 – 6.22	0.309												
DEHSI	-11.40	-28.22 – 5.43	0.184												
Number of brain injuries															
0	Ref														
1	1.01	-3.65 – 5.67	0.670												
2	-7.70	-13.66 – -1.74	0.011												
3	-17.96	-29.44 – -6.49	0.002												
4	-27.40	-42.39 – -12.42	<0.001												
5	-19.90	-22.57 – -17.24	<0.001												
6	-14.90	-17.57 – -12.24	<0.001												
>1 brain injury	-11.88	-17.14 – -6.62	<0.001							-6.92	-13.99 – 0.15	0.055			
Severe brain injuries	-8.97	-15.24 – -2.70	0.005												

Number of severe brain injuries															
0	Ref														
1	-3.72	-10.68 – 3.25	0.295												
2	-25.87	-36.55 – -15.19	<0.001												
3	-17.19	-35.81 – 1.44	0.070												
5	-14.19	-16.36 – -12.01	<0.001												
>1 severe brain injury	-21.93	-31.07 – -12.78	<0.001							-18.35	-35.91 – -0.78	0.041	-10.30	-20.26 – -0.33	0.043

Brain injury: IVHI°-III°, PVHI, moderate/severe ventricular dilatation, CBH, punctate white matter lesions, cPVL, *BPD:* bronchopulmonary dysplasia, *CBH:* cerebellar hemorrhage, *CPAP:* continuous positive airway pressure, *cPVL:* cystic periventricular leukomalacia, *DEHSI:* diffuse excessive high signal intensity, *IVH:* intraventricular hemorrhage, *NEC:* necrotizing enterocolitis, *PDA:* patent ductus arteriosus, *PPROM:* preterm premature rupture of membranes, *PVHI:* periventricular hemorrhagic infarction, *ROP:* retinopathy of prematurity, severe brain injury: IVH III°, PVHI, CBH III°+IV°, severe ventricular dilatation, cPVL, *SGA:* small for gestational age, *transfusion of RBCs:* transfusion of red blood cells, significant p:<0.05.* Variable was excluded after regression diagnostics.
